# A New Adaptive H-Infinity Filtering Algorithm for the GPS/INS Integrated Navigation

**DOI:** 10.3390/s16122127

**Published:** 2016-12-19

**Authors:** Chen Jiang, Shu-Bi Zhang, Qiu-Zhao Zhang

**Affiliations:** 1 School of Environment Science and Spatial Informatics, China University of Mining and Technology, Xuzhou 221116, China; jiangchen@cumt.edu.cn (C.J.); qiuzhao.zhang@cumt.edu.cn (Q.-Z.Z.); 2 Collaborative Innovation Center for Resource Utilization and Ecological Restoration of Old Industrial Base, China University of Mining and Technology, Xuzhou 221116, China

**Keywords:** adaptive Kalman filter, H-infinity filter, integrated navigation, robust estimation

## Abstract

The Kalman filter is an optimal estimator with numerous applications in technology, especially in systems with Gaussian distributed noise. Moreover, the adaptive Kalman filtering algorithms, based on the Kalman filter, can control the influence of dynamic model errors. In contrast to the adaptive Kalman filtering algorithms, the H-infinity filter is able to address the interference of the stochastic model by minimization of the worst-case estimation error. In this paper, a novel adaptive H-infinity filtering algorithm, which integrates the adaptive Kalman filter and the H-infinity filter in order to perform a comprehensive filtering algorithm, is presented. In the proposed algorithm, a robust estimation method is employed to control the influence of outliers. In order to verify the proposed algorithm, experiments with real data of the Global Positioning System (GPS) and Inertial Navigation System (INS) integrated navigation, were conducted. The experimental results have shown that the proposed algorithm has multiple advantages compared to the other filtering algorithms.

## 1. Introduction

Accurate Global Positioning System (GPS) measurement data can be used to control the Inertial Navigation System (INS) accumulative errors. On the other hand, the INS can be used in cases when the GPS fails. Thus, the integration of GPS and INS has been widely adopted in the field of dynamic navigation and positioning. The Kalman filter is one of the most celebrated real-time optimal estimators [1], and therefore it has become the basic data-fusion approach in navigation and positioning [2,3]. Unfortunately, the Kalman filter performance depends on many factors, thus it cannot meet the requirements of certain nonlinear systems and the performance is susceptible to outliers. Hence, many improved filtering algorithms were proposed, for instance, the unscented Kalman filter [4,5], the particle filter [6], the robust filter [7,8], the adaptive filter [9,10,11,12], and so forth. The nonlinear problem can be handled by the unscented Kalman filter or the particle filter, however, they both become unstable in a high-dimensional case. On the other hand, the cubature Kalman filter is a recently developed nonlinear filtering algorithm [13]. Compared with the unscented Kalman filter, the cubature Kalman filter shows better performance in stability; therefore, it has been adopted in the GPS/inertial measurement unit (IMU) integrated navigation system [14]. Many forms of robust filtering algorithms were proposed to control the influence of outliers, but the influence of the model errors was neglected. The multiple-model-based adaptive estimation (MMAE) and innovation-based adaptive estimation (IAE) represent the adaptive Kalman filtering approaches. In the MMAE, there is a set of Kalman filters which work in parallel under different models, while in the IAE the adaptation is applied directly to the statistical information. Moreover, the IAE is more applicable to the GPS/INS integrated navigation systems [9]. The DIA (detection, identification, and adaptation) methods represent the recursive testing procedures, and they were proposed to eliminate the effects of outliers [15]. In the DIA methods, firstly, the model errors are detected and identified, then deviations of the state estimates caused by the model errors are eliminated, and finally, the adaptation in which the model is recovered from the identified errors, is implemented. However, the identification is quite difficult, especially when the measurements are not accurate [16].

Recently, the adaptively robust filtering has been proposed for dynamic navigation and positioning [16,17]. The combination of adaptive filter and robust filter provides the required adaptivity and robustness, and the adaptive–robust filter has a better ability to mitigate the influences of dynamic model deviations and outlying measurements [16,18,19]. Nevertheless, the number of the measurements should be equal to or greater than the state vector dimension. Hence, the adaptively robust filtering can be applied in a limited number of cases [20]. It should be noticed that the aforementioned filtering algorithms do not consider the interference. The minimization of estimation error was used to develop the H-infinity filter without the information on statistical properties [21,22]. The H-infinity filter can be used to address the uncertainties in the measurement noise [20,23]. Hassibi has shown that the least mean squares (LMS)-based adaptive filtering algorithm represents the H-infinity optimal, and that the H-infinity algorithm is applicable in the uncertain environments [24]. However, the median-based filters might be highly robust, but not efficient [20]. Moreover, the H-infinity filter fails in the presence of outliers [8]. The performance of the H-infinity filter can be improved only if the effects of the outliers are controlled. The robust estimation method provides a way to control the influence of outliers [25,26,27]. Hence, the robust estimation method can be applied to improve the robustness and stability of the H-infinity filter. Accordingly, in order to control the outliers influence, a new filtering scheme based on combination of the adaptive filter and the H-infinity filter should be developed.

This paper focuses on a comprehensive filtering algorithm based on the combination of the adaptive filter and the H-infinity filter. According to the model errors and interference, a novel filtering algorithm intended for the GPS/INS integrated navigation is developed. The GPS/INS integrated dynamic navigation was tested using the cubature Kalman filter. The experimental results have shown that the proposed algorithm has better performance compared to the commonly used filtering algorithms.

The paper is organized as follows. In Section 2, a basic principle of the adaptive filter and the H-infinity filtering algorithms are introduced, and scaling factors are discussed. In Section 3, the adaptive H-infinity filtering algorithm and the robust estimation method are presented. The equations of dynamic model and measurement model intended for the GPS/INS integrated navigation are listed in Section 4. In Section 5, the proposed algorithm is verified by experiments and the experimental results are provided; in addition, a comparison to other filtering algorithms is performed and the obtained results are discussed. Lastly, a brief conclusion is given in Section 6.

## 2. The Adaptive Kalman Filter and the H-Infinity Filter

### 2.1. The Adaptive Kalman Filter

Various filtering algorithms have been developed in order to mitigate the influence of model errors, such as the adaptive Kalman filter. The adaptive algorithms intended for the GPS/INS integrated navigation can be divided into multiple-model adaptive estimation, adaptive stochastic modeling, and covariance scaling [28]. In the multiple-model adaptive estimation, a set of Kalman filters works in parallel, under different models of the filter’s statistical information [9]. A single or multiple factor(s) of the state covariance matrix can be used to improve the stability and convergence performance, thus, it is important to select a suitable scaling factor. In general, the scaling factor is determined empirically, based on experience or innovation and residual information [23]. In reference [12], two adaptive factors were derived, and the filtering performance of the algorithm based on the optimal adaptive factor was superior to the one based on the nonoptimal adaptive factors.

The dynamic model and measurement model are given by:

(1) $$\left\{ \begin{array}{l} x_{k}=\Phi_{k,k-1}x_{k-1}+w_{k}\\ z_{k}=H_{k}x_{k}+v_{k} \end{array}\right.,$$

where $x_{k}$ is the state vector, $\Phi_{k,k-1}$ presents the state transition matrix, $H_{k}$ is the measurement matrix, $z_{k}$ denotes the measurement vector, and $w_{k}$ and $v_{k}$ are the system noise and measurement noise, respectively. The predicted state vector and its covariance matrix are defined by:

(2) $$x_{k/k-1}=\Phi_{k,k-1}x_{k-1},$$

(3) $$P_{k/k-1}=\Phi_{k,k-1}P_{k-1}{\Phi^{T}}_{k,k-1}+Q_{k},$$

where $x_{k/k-1}$ is the predicted state vector, and $P_{k/k-1}$ and $Q_{k}$ denote the predicted covariance matrices of $x_{k/k-1}$ and $w_{k}$, respectively.

According to the rule of the least squares estimation, the following can be written:

(4) $$V_{k}^{T}R_{k}^{-1}V_{k}+V_{x_{k/k-1}}^{T}P_{k/k-1}^{-1}V_{x_{k/k-1}}=\min,$$

where $V_{k}$ and $V_{x_{k/k-1}}$ denote the residual vectors of $z_{k}$ and $x_{k/k-1}$, respectively.

The solution of the standard Kalman filter is obtained by:

(5) $$K_{k}=P_{k/k-1}H_{k}^{T}{\left(H_{k}P_{k/k-1}H_{k}^{T}+R_{k}\right)}^{-1},$$

(6) $$x_{k/k}=x_{k/k-1}+K_{k}(z_{k}-H_{k}x_{k/k-1}),$$

where $R_{k}$ is the covariance matrix of the measurement noise. If the risk function is defined by:

(7) $$V_{k}^{T}R_{k}^{-1}V_{k}+\alpha_{k}V_{x_{k/k-1}}^{T}P_{k/k-1}^{-1}V_{x_{k/k-1}}=\min,$$

then the solution of the adaptive Kalman filter is obtained by:

(8) $$x_{k/k}={\left(H_{k}^{T}R_{k}^{-1}H_{k}+\alpha_{k}P_{k/k-1}^{-1}\right)}^{-1}(H_{k}^{T}R_{k}^{-1}z_{k}+\alpha_{k}P_{k/k-1}^{-1}x_{k/k-1}),$$

where $\alpha_{k}$ denotes the adaptive factor. The equivalent expression of Equation (8) is in reference [27]:

(9) $$x_{k/k}=x_{k/k-1}+\frac{1}{\alpha_{k}}P_{k/k-1}H_{k}^{T}(\frac{1}{\alpha_{k}}H_{k}P_{k/k-1}H_{k}^{T}+R_{k})(z_{k}-H_{k}x_{k/k-1}),$$

Four types of the adaptive factors and error-learning statistics were summarized in reference [17]. The predicted residual statistic was adopted if no redundant information exists, and the adaptive factor based on the three-segment function was [16,29]:

(10) $$\alpha_{k}=\left\{ \begin{array}{ll} 1 & \;\left|\Delta {\bar{V}}_{k}\right|\leq c_{0}\\ \frac{c_{0}}{\left|\Delta {\bar{V}}_{k}\right|}{\left(\frac{c_{1}-\left|\Delta {\bar{V}}_{k}\right|}{c_{1}-c_{0}}\right)}^{2} & c_{0}<\left|\Delta {\bar{V}}_{k}\right|\leq c_{1}\\ 0 & \;\left|\Delta {\bar{V}}_{k}\right|>c_{1} \end{array}\right.,$$

where $\Delta {\bar{V}}_{k}$ denotes the learning statistic based on the predicted residual, $\Delta {\bar{V}}_{k}={\left(\frac{{\bar{V}}_{k}^{T}{\bar{V}}_{k}}{\mathrm{tr}(P_{{\bar{V}}_{k}})}\right)}^{\frac{1}{2}}$, $P_{{\bar{V}}_{k}}=H_{k}P_{k/k-1}H_{k}^{T}+R_{k}$, ${\bar{V}}_{k}$ denotes the innovation vector, further, $1.0\leq c_{0}\leq 1.5$, and $3.0\leq c_{1}\leq 8.5$. In Equation (9), $\alpha_{k}\neq 0$, therefore, when Equation (9) is used, the adaptive factor based on the two-segment function [17], defined by Equation (11), should be adopted:

(11) $$\alpha_{k}=\left\{ \begin{array}{ll} 1 & \left|\Delta {\bar{V}}_{k}\right|\leq c\\ \frac{c}{\left|\Delta {\bar{V}}_{k}\right|} & \left|\Delta {\bar{V}}_{k}\right|>c \end{array}\right.,$$

where 1.0≤c≤2.5, and the optimal value of c is 1.0 [12,17].

### 2.2. Basic Principle of the H-Infinity Filter

The H-infinity filter is a special form of the Kalman filter, and the principle of the H-infinity filter is based on the H-infinity optimal estimation that guarantees the smallest estimation energy error for all possible disturbances of the fixed energy [30]. The cost function of a nonlinear filter is defined by:

(12) $$J=\frac{\sum_{k=1}^{N}{\left\|x_{k}-{\hat{x}}_{k}\right\|}^{2}}{{\left\|x_{0}-{\hat{x}}_{0}\right\|}_{P_{0}^{-1}}^{2}+\sum_{k=1}^{N}\left({\left\|w_{k}\right\|}_{Q_{k}^{-1}}^{2}+{\left\|v_{k}\right\|}_{R_{k}^{-1}}^{2}\right)},$$

where N is the number of filtering epochs, $x_{0}$ is the initial value of the state vector x with the covariance matrix $P_{0}$, ${\hat{x}}_{0}$ and ${\hat{x}}_{k}$ are the estimated state vectors of $x_{0}$ and $x_{k}$, respectively, and the expression ${\left\|x_{0}-{\hat{x}}_{0}\right\|}_{P_{0}^{-1}}^{2}$ denotes ${\left(x_{0}-{\hat{x}}_{0}\right)}^{T}P_{0}^{-1}(x_{0}-{\hat{x}}_{0})$. In general, the estimate, ${\hat{x}}_{k}$, which satisfies ${\hat{x}}_{k}=\arg\;\min{\left\|J\right\|}_{\infty }$, should be found in order to minimizes J. Actually, an analytical solution for the optimal H-infinity filter is difficult to achieve. Thus, a suboptimal recursion algorithm is developed by setting a threshold value, γ, which satisfies the following Riccati inequality [31]:

(13) $$P_{k}^{-1}+H_{k}^{T}H_{k}-\gamma^{2}L_{k}^{T}L_{k}>0,$$

where $P_{k}$ is the covariance matrix of $x_{k}$, $L_{k}$ is the coefficient matrix of the constraint equation, and $L_{k}$ is generally defined by the unit matrix I. The recursion equations of the H-infinity filter are listed below [31]:

(14) $$x_{k/k-1}=\Phi_{k,k-1}x_{k-1},$$

(15) $$P_{k/k-1}=\Phi_{k,k-1}P_{k-1/k-1}\Phi_{k,k-1}^{T}+Q_{k},$$

(16) $$x_{k/k}=x_{k/k-1}+K_{k}(z_{k}-H_{k}x_{k/k-1}),$$

(17) $$K_{k}=P_{k/k-1}H_{k}^{T}{\left(H_{k}P_{k/k-1}H_{k}^{T}+R_{k}\right)}^{-1},$$

(18) $$P_{k/k}=P_{k/k-1}-\Phi_{k/k-1}P_{k/k-1}[H_{k}^{T}\;L_{k}^{T}]R_{e,k}^{-1}\left[\begin{array}{l} H_{k}\\ L_{k} \end{array}\right]P_{k/k-1}\Phi_{k/k-1}^{T},$$

(19) $$R_{e,k}^{-1}=\left[\begin{array}{l} I\;0\\ 0\;-\gamma^{2}I \end{array}\right]+\left[\begin{array}{l} H_{k}\\ L_{k} \end{array}\right]P_{k/k-1}[H_{k}^{T}\;L_{k}^{T}],$$

The H-infinity filter minimizes the estimation error in the worst case, which makes it more robust than the standard Kalman filter. In addition, the H-infinity filter becomes more robust when the constraint parameter γ decreases. However, the value of γ should not be in the vicinity of zero, because that might cause the divergence of the H-infinity filter [32]. In general, γ is fixed to certain value, which is chosen by experience. In addition, the H-infinity filter represents a rigorous method for the system with an unreliable model [30].

## 3. A Novel Adaptive H-Infinity Filtering Algorithm

In the adaptive filtering algorithms, it is assumed that the measurements are reliable and that the predicted residual can reflect the deviations of the model information. However, some of the adaptive filtering algorithms cannot control the effects of the outliers. As mentioned before, the H-infinity filter can overcome the uncertainties in the measurements, but the effects of the outliers cannot be controlled just by the H-infinity filter, thus, some robust Kalman filtering algorithms were developed [7].

In this paper, an integrated adaptive H-infinity filtering algorithm is proposed. An adaptive factor was constructed in order to control the influence of the dynamic model errors, and the H-infinity filter was adopted to address the uncertain interference. The recursion equations of the adaptive H-infinity filter can be expressed by:

(20) $$x_{k/k-1}=\Phi_{k,k-1}x_{k-1},$$

(21) $$P_{k/k-1}=\Phi_{k,k-1}P_{k-1/k-1}\Phi_{k,k-1}^{T}+Q_{k},$$

(22) $$x_{k/k}=x_{k/k-1}+{\bar{K}}_{k}(z_{k}-H_{k}x_{k/k-1}),$$

(23) $${\bar{K}}_{k}=\frac{1}{\alpha_{k}}P_{k/k-1}H_{k}^{T}{\left(\frac{1}{\alpha_{k}}H_{k}P_{k/k-1}H_{k}^{T}+R_{k}\right)}^{-1},$$

(24) $$P_{k/k}=\frac{1}{\alpha_{k}}P_{k/k-1}-\frac{1}{\alpha_{k}^{2}}\Phi_{k/k-1}P_{k/k-1}[H_{k}^{T}\;L_{k}^{T}]{\bar{R}}_{e,k}^{-1}\left[\begin{array}{l} H_{k}\\ L_{k} \end{array}\right]P_{k/k-1}\Phi_{k/k-1}^{T},$$

(25) $${\bar{R}}_{e,k}^{-1}=\left[\begin{array}{l} I\;0\\ 0\;-\gamma^{2}I \end{array}\right]+\frac{1}{\alpha_{k}}\left[\begin{array}{l} H_{k}\\ L_{k} \end{array}\right]P_{k/k-1}[H_{k}^{T}\;L_{k}^{T}],$$

where ${\bar{K}}_{k}$ denotes the equivalent gain matrix of the H-infinity filter. Since, there were no redundant measurements, the adaptive factor based on the two-segment function was adopted, and the predicted residual was selected as the statistic value.

In the integrated adaptive H-infinity filtering algorithm, the robust estimation method was employed to decrease the effects of outlying measurements. The selection of the equivalent weight and equivalent covariance matrix should be considered in the robust estimation. In general, the components of the predicted state vector are correlated, so the equivalent covariance matrix constructed by scaling factor, $\lambda_{ij}$, is applied. The scaling factor is defined by reference [33]:

(26) $$\lambda_{ij}=\sqrt{\lambda_{ii}}\sqrt{\lambda_{jj}},$$

(27) $$\lambda_{ii}=\left\{ \begin{array}{ll} 1 & \left|{\bar{V}}_{{\bar{X}}_{k_{i}}}\right|\leq c\\ \frac{\left|{\bar{V}}_{{\bar{X}}_{k_{i}}}\right|}{c} & \left|{\bar{V}}_{{\bar{X}}_{k_{i}}}\right|>c \end{array}\right.,$$

where $\left|{\bar{V}}_{{\bar{X}}_{k_{i}}}\right|$ denotes the component of the predicted residual vector, 1.0≤c≤1.5, and $\lambda_{jj}$ can be expressed the same as $\lambda_{ii}$. Thus, the equivalent covariance matrix ${\bar{R}}_{k}$ is obtained by

(28) $${\bar{R}}_{k}=\lambda_{ij}R_{k},$$

**Figure 1:**
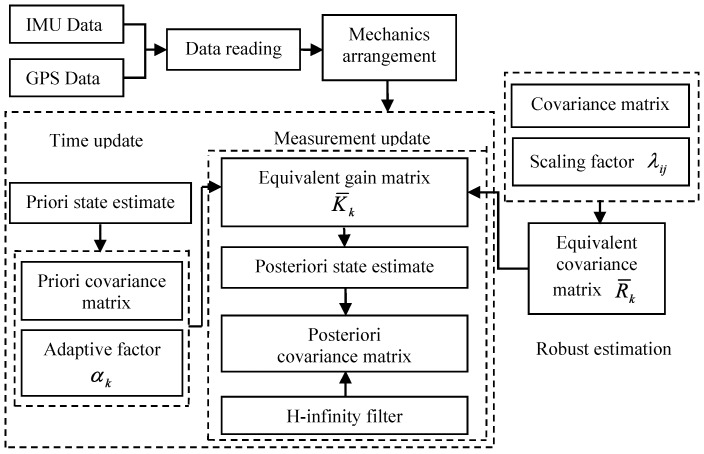
A flow chart of the adaptive H-infinity algorithm.

The entire process of the proposed filtering algorithm is shown in Figure 1.

## 4. The GPS/INS Integrated Navigation

Recently, in the GPS/INS integrated navigation, three types of integration have been developed: loosely coupled, tightly coupled, and deeply coupled [34]. In the loosely coupled integrated navigation, a 15-dimension state vector is adopted, which includes the deviations of position, velocities, attitudes, and the noises of gyroscope and accelerometer. Thus, the state vector $\hat{x}$ is defined by:

(29) $$\hat{x}=[\delta x,\delta y,\delta z,\delta v_{x},\delta v_{y},\delta v_{z},\delta \phi_{e},\delta \phi_{n},\delta \phi_{u},\delta g_{x},\delta g_{y},\delta g_{z},\delta a_{x},\delta a_{y},\delta a_{z}],$$

Since, the GPS/INS integrated navigation system represents a nonlinear system, the cubature Kalman filter is applied to address the nonlinear problem. For a discrete nonlinear system, it can be written as follows:

(30) $$\left\{ \begin{array}{l} x_{k}=f(x_{k-1})+w_{k}\\ z_{k}=h(x_{k})+v_{k} \end{array}\right.,$$

where f(⋅) and h(⋅) denote the known nonlinear functions. The cubature points are generated by:

(31) $$\xi =\sqrt{\frac{m}{2}}{\left[1\right]}_{i},\;i=1,\ldots ,m,$$

where m denotes the number of the cubature points and m=2n, n denotes the dimension of the state vector, and [1] denotes the point $\left(\begin{array}{l} 1\\ 0 \end{array}\right)$ in this paper. Then, the equations of the cubature Kalman filter are given by reference [14]:

(a) Time update

(32) $$\left\{ \begin{array}{l} S_{k-1/k-1}=SVD(P_{k-1/k-1})\\ X_{k-1,k-1}=S_{k-1/k-1}\xi +x_{k-1/k-1}\\ X_{k/k-1}^{*}=f(X_{k-1,k-1}) \end{array}\right.,$$

(33) $$\left\{ \begin{array}{l} x_{k/k-1}=\frac{1}{m}\sum_{i=1}^{m}X_{i,k/k-1}^{*}\\ P_{k/k-1}=\frac{1}{m}\sum_{i=1}^{m}X_{i,k/k-1}^{*}X_{i,k/k-1}^{*T}-x_{k/k-1}x_{k/k-1}^{T}+Q_{k} \end{array}\right.,$$

(b) Measurement update

(34) $$\left\{ \begin{array}{l} s_{k/k-1}=SVD(P_{k/k-1})\\ X_{k/k-1}=s_{k/k-1}\xi +x_{k/k-1}\\ Y_{k/k-1}=h(X_{i,k/k-1})\\ y_{k/k-1}=\frac{1}{m}\sum_{i=1}^{m}Y_{i,k/k-1}\\ P_{zz,k/k-1}=\frac{1}{m}\sum_{i=1}^{m}Y_{i,k/k-1}Y_{i,k/k-1}^{T}-y_{k/k-1}y_{k/k-1}^{T}+R_{k}\\ P_{xz,k/k-1}=\frac{1}{m}\sum_{i=1}^{m}X_{i,k/k-1}Y_{i,k/k-1}^{T}-x_{k/k-1}y_{k/k-1}^{T} \end{array}\right.,$$

then the final measurement update equations are obtained by:

(35) $$\left\{ \begin{array}{l} x_{k/k}=x_{k/k-1}+K_{k}(z_{k}-z_{k/k-1})\\ K_{k}=P_{xz,k/k-1}P_{zz,k/k-1}^{-1}\\ P_{k/k}=P_{k/k-1}-K_{k}P_{zz,k/k-1}K_{k}^{T} \end{array}\right.,$$

where S denotes the square root of the covariance matrix P, $X_{k-1,k-1}$ denotes the cubature points of the states vector, $X_{k/k-1}^{*}$ denotes the propagated cubature points, “SVD” denotes the singular value decomposition of a matrix; and $Z_{k/k-1}$ denotes the propagated cubature points of the measurement vector. Accordingly, the adaptive H-infinity filtering algorithm intended for the GPS/INS integrated navigation system is summarized as follows:

(a) Time update

(36) $$x_{k/k-1}=\frac{1}{m}\sum_{i=1}^{m}X_{i,k/k-1}^{*},$$

(37) $$P_{k/k-1}=\frac{1}{m}\sum_{i=1}^{m}X_{i,k/k-1}^{*}X_{i,k/k-1}^{*T}-x_{k/k-1}x_{k/k-1}^{T}+Q_{k},$$

(b) Measurement update

(38) $$x_{k/k}=x_{k/k-1}+K_{k}(z_{k}-z_{k/k-1}),$$

(39) $$K_{k}=P_{xz,k/k-1}P_{zz,k/k-1}^{-1},$$

(40) $$P_{k/k}=\frac{1}{\alpha_{k}}P_{k/k-1}-[P_{xz,k}\;\frac{1}{\alpha_{k}}P_{k/k-1}]{\left[\begin{array}{ll} P_{zz,k}-R_{k}+I & P_{xz,k}^{T}\\ P_{xz,k} & \frac{1}{\alpha_{k}}P_{k/k-1}-\gamma^{2}I \end{array}\right]}^{-1}\left[\begin{array}{l} P_{xz,k}^{T}\\ {\left(\frac{1}{\alpha_{k}}P_{k/k-1}\right)}^{T} \end{array}\right],$$

In addition, $R_{k}$ should be replaced with the equivalent covariance matrix ${\bar{R}}_{k}$ in order to make the algorithm more robust.

In the loosely coupled integrated navigation, the differences in position and velocity, $Z_{\rho }$, between GPS and INS are considered as the external measurements, namely:

(41) $$Z_{\rho }(t)=\rho_{\mathrm{GPS}}-\rho_{\mathrm{INS}},$$

where $\rho_{\mathrm{GPS}}$, namely, $x_{\mathrm{GPS}}$, $y_{\mathrm{GPS}}$, $z_{\mathrm{GPS}}$, $v_{\mathrm{XGPS}}$, $v_{\mathrm{YGPS}}$, and $v_{\mathrm{ZGPS}}$, presents the output information of the GPS in the WGS-84 coordinate system, while the output information of the INS is $\rho_{\mathrm{INS}}$, namely, $x_{\mathrm{INS}}$, $y_{\mathrm{INS}}$, $z_{\mathrm{INS}}$, $v_{\mathrm{XINS}}$, $v_{\mathrm{YINS}}$, and $v_{\mathrm{ZINS}}$. Then, the measurement equation is as follows:

(42) $$Z_{k}=\left[\begin{array}{l} r_{\mathrm{GPS}}-r_{\mathrm{INS}}\\ v_{\mathrm{GPS}}-v_{\mathrm{INS}} \end{array}\right],$$

where $Z_{k}$ represents the measurement vector of the integrated system, and $r_{\mathrm{GPS}}$, $r_{\mathrm{INS}}$ and $v_{\mathrm{GPS}}$, $v_{\mathrm{INS}}$ are position and velocity output information of GPS and INS, respectively. Compared to the loosely coupled, the tightly coupled integration has better performance in terms of precision. However, the loosely coupled navigation can be implemented easily, and the computation process is more concise [35].

## 5. Test Cases and Data Analysis

### 5.1. Test Case 1

**Figure 2:**
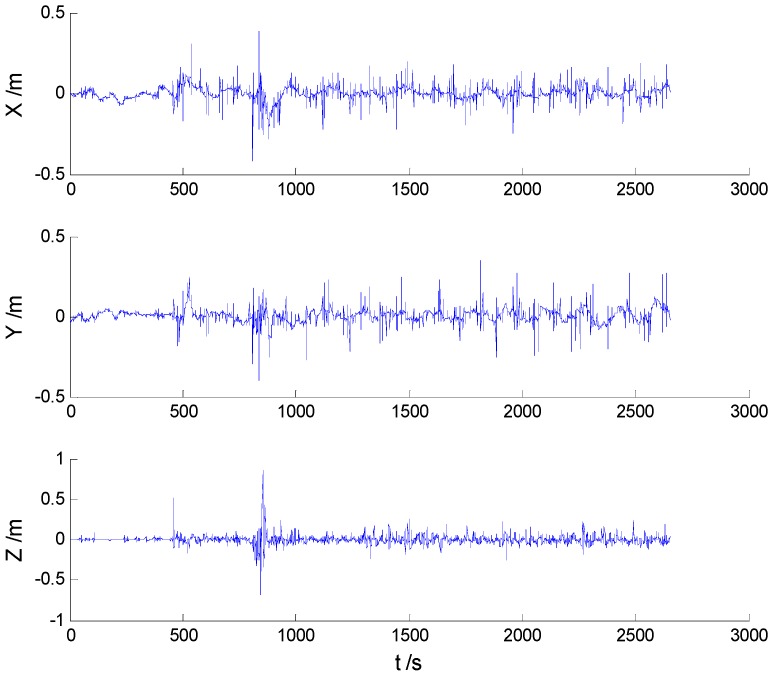
Position errors of the Kalman filter (KF) algorithm.

**Figure 3:**
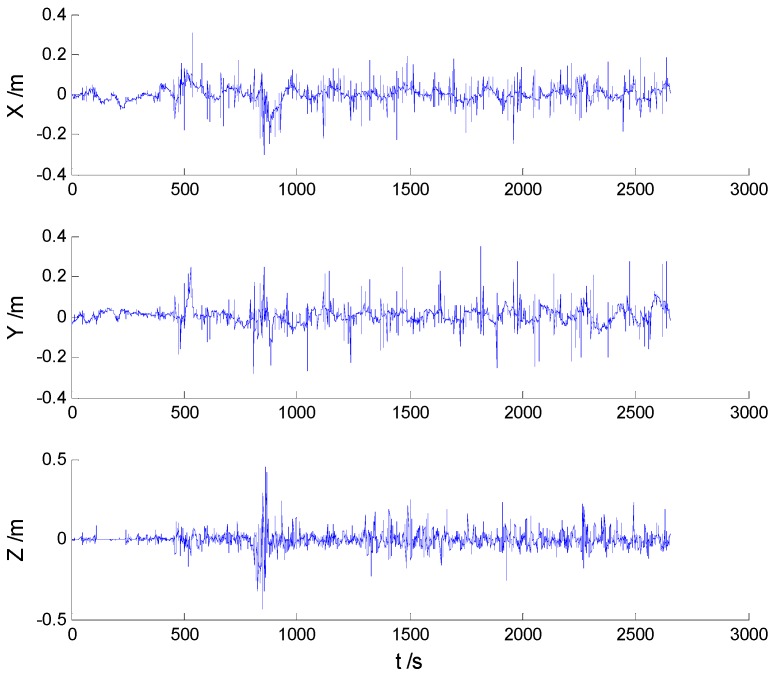
Position errors of the adaptive Kalman filter (AKF) algorithm.

**Figure 4:**
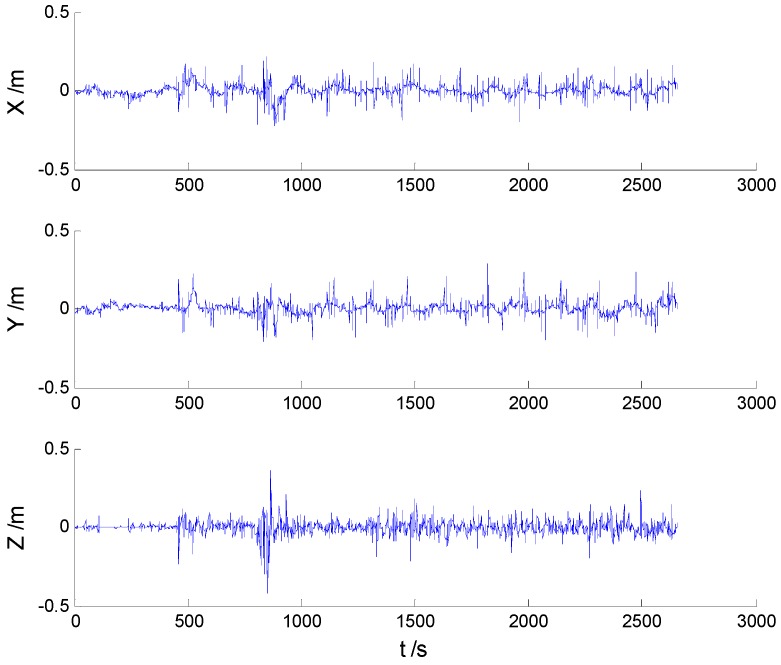
Position errors of the H-infinity filter (HF) algorithm.

**Figure 5:**
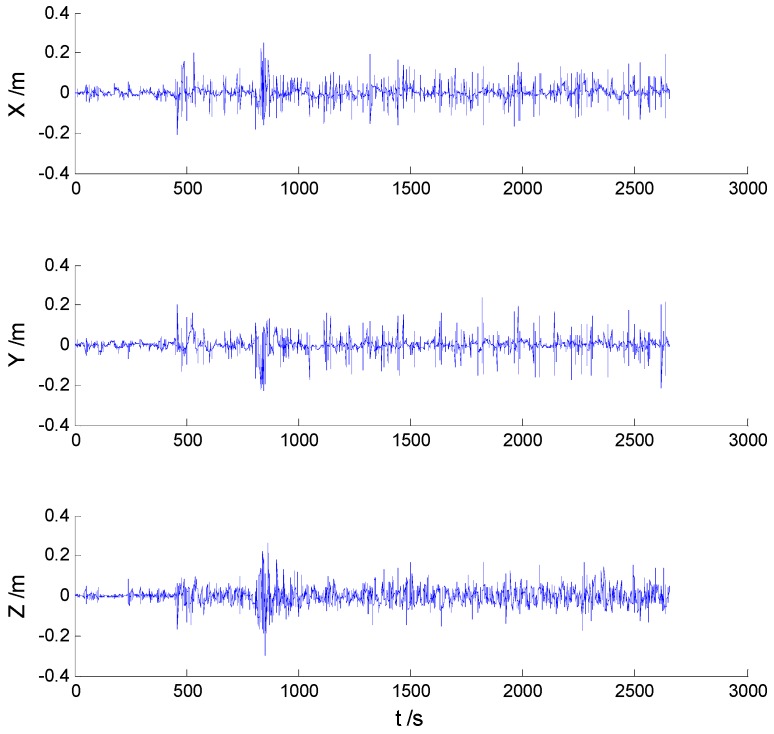
Position errors of the adaptive H-infinity filter (AHF) algorithm.

In this case, the GPS and INS data were obtained by a small aerial vehicle equipped with inertial sensors of automotive-grade quality and a GPS receiver [36]. Four schemes were implemented to examine the performance of the proposed filtering algorithm:

Scheme 1: Kalman Filter (KF);

Scheme 2: Adaptive Kalman Filter (AKF) (two-segment function was adopted and c=1.0);

Scheme 3: H-infinity Filter (HF);

Scheme 4: Adaptive H-infinity Filter (AHF) (two-segment function was adopted and c=1.0, and the robust estimation method was adopted with the double-factors and $c^{\prime }=1.5$, where $c^{\prime }$ denotes the criterion of the double-factors equivalent covariance matrix);

Position errors of all mentioned schemes are demonstrated in the following:

As it can be seen in previous figures, there is little difference between Figure 2 and Figure 3, which indicates that there are limited effects of the model errors. Due to the minimization of the worst-case estimation error, the H-infinity filter performs slightly better than the adaptive Kalman filter, which can be seen from Figure 4 and Table 1. Nonetheless, Figure 5 indicates that a noticeable improvement was obtained by the adaptive H-infinity filtering algorithm in terms of dynamic model errors and the uncertain interferences.

The Root Mean Squares Errors (RMSEs) of the schemes are presented in Table 1.

### 5.2. Test Case 2

In this experiment, data were collected by a vehicle equipped with a GPS/INS integrated navigation system, which was composed of two GPS receivers and an inertial measurement unit (IMU). One of the GPS receivers was set as a reference station, and another receiver as well as the IMU were mounted on the vehicle. GPS data were calculated by the double difference pseudorange with variances of 0.25 m^2^ and 0.0025 m^2^/s^2^, respectively. The sampling frequencies of GPS and IMU were 1 Hz and 100 Hz, respectively. The precise results achieved by the double difference carrier phase were regarded as references. Afterwards, four schemes were performed in the integrated navigation system:

Scheme 1: Kalman Filter (KF);

Scheme 2: Adaptive Kalman Filter (AKF) (two-segment function was adopted and c=1.0);

Scheme 3: H-infinity Filter (HF);

Scheme 4: Adaptive H-infinity Filter (AHF) (two-segment function was adopted and c=1.0, and the robust estimation method was adopted with the double-factors where $c^{\prime }=1.5$).

#### 5.2.1. Experiments without Outliers

**Figure 6:**
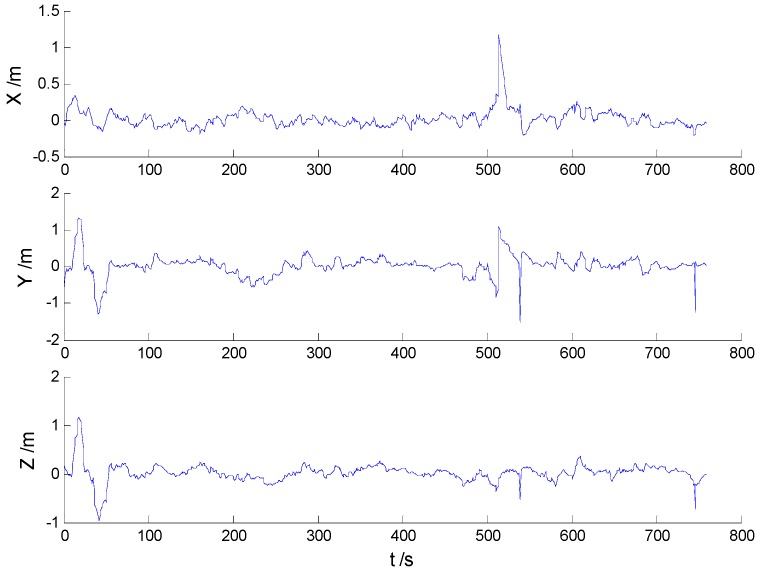
Position errors of the KF algorithm.

**Figure 7:**
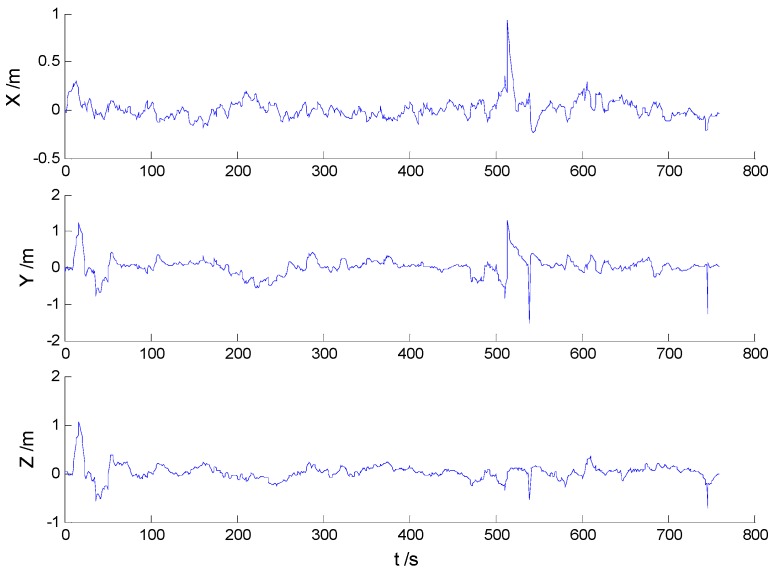
Position errors of the AKF algorithm.

**Figure 8:**
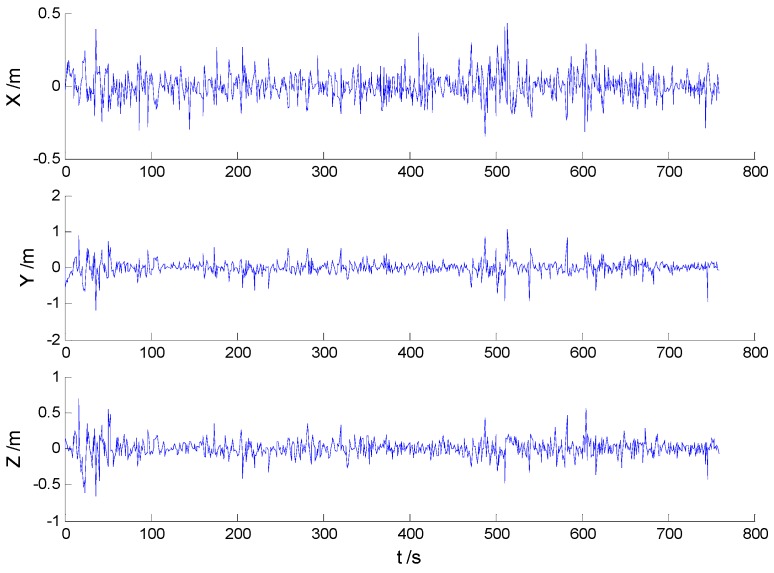
Position errors of the HF algorithm.

**Figure 9:**
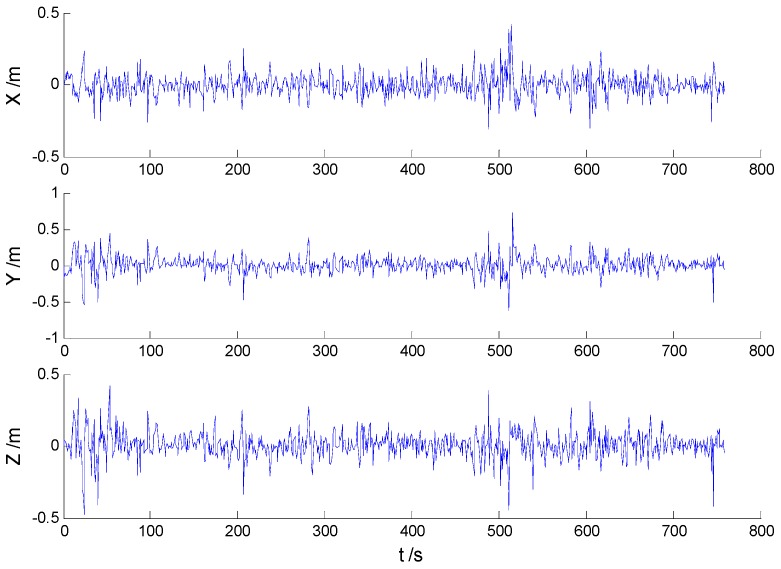
Position errors of the AHF algorithm.

Position errors of used schemes are demonstrated in the following:

Due to the vehicle’s movement over the bumps, disturbances can be found in both Figure 6 and Figure 7, which indicates that the robustness of the KF and AKF algorithms should be improved. Because of decrease of the dynamic model errors, the AKF algorithm shows a slightly better performance than the KF algorithm. Apparently, the uncertain interference is controlled, thus, the HF and AHF algorithms (Figure 8 and Figure 9) perform much better than the first two algorithms. The robustness of the AHF algorithm is improved by the robust estimation method, therefore, the error amplitude is reduced. The RMSE of each scheme is given in Table 2.

As mentioned before, RMSEs of the AKF and HF algorithms, presented in Table 2, are both smaller than RMSE of the KF algorithm. The integration of the AKF and HF algorithms into the AHF algorithm provides better performance compared to the other algorithms.

#### 5.2.2. Experiments with Outliers

**Figure 10:**
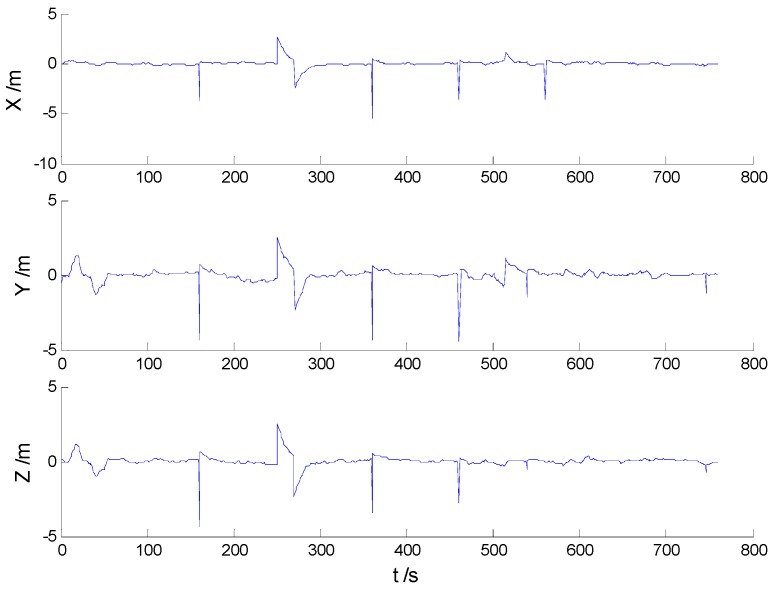
Position errors of the KF algorithm.

**Figure 11:**
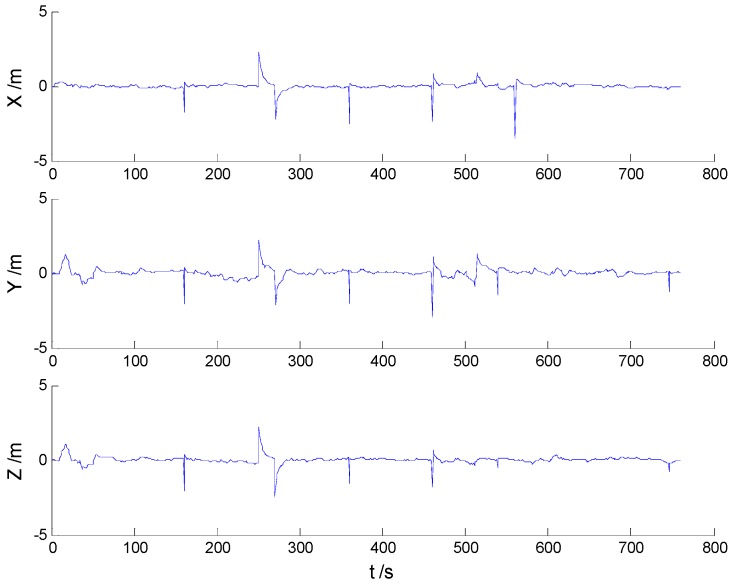
Position errors of the AKF algorithm.

**Figure 12:**
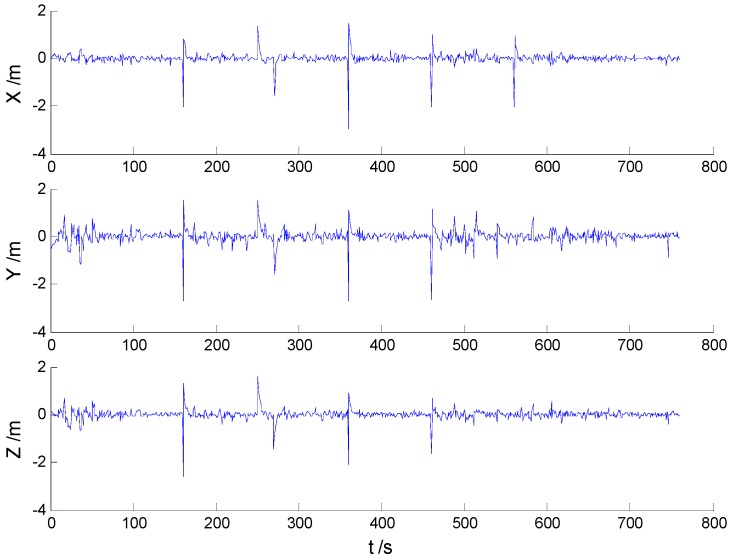
Position errors of the HF algorithm.

**Figure 13:**
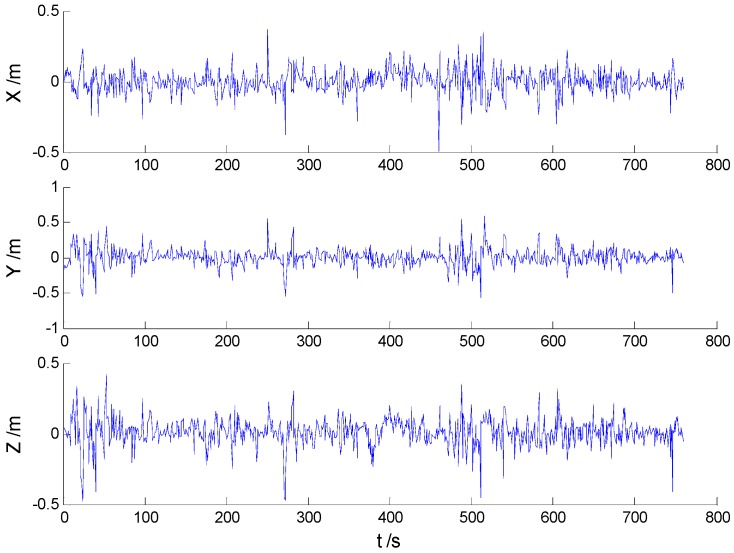
Position errors of the AHF algorithm.

In these experiments, we used data from the Section 5.2.1, but the single gross errors were added artificially to the GPS measurement data at the 160th, 360th, 460th, and 560th epochs, respectively, and the constantly changed outliers were added to all epochs between the 251th and 280th epochs. Then, the previously used schemes were implemented again, and the obtained position errors of all schemes are displayed in Figure 10, Figure 11, Figure 12 and Figure 13.

According to the presented results, it can be concluded that in the presence of outliers, filtering results were mostly dependent on outliers. Moreover, Figure 10 and Figure 11 show that the KF and AKF filtering results have similar trends, while the latter figure manifests a reduced error. As it can be seen in Figure 12, the filtering results of the HF algorithm are stable except in the epochs that contain outliers. Furthermore, Figure 13 shows that the proposed filtering algorithm has better performance than other algorithms, so the influence of single and constantly changed outliers is reduced using the AHF algorithm. The RMSE values of all used scheme are presented in Table 3.

Compared to the Kalman filter, the filtering precision of the AKF and HF algorithms is improved. However, both AKF and HF algorithms are significantly affected by the outliers. On the other hand, the AHF algorithm shows better fault tolerance and robustness compared to other schemes. The adaptive H-infinity filter is more robust because of the robust estimation method, based on the control of dynamic model errors and uncertain interference. In all presented cases, RMSEs of the AHF algorithm are the smallest for all coordinates, which means that the positions calculated by the AHF algorithm are in good agreement with the actual positions.

## 6. Conclusions

An integrated adaptive H-infinity filtering algorithm for mitigation of positioning errors caused by dynamic model errors and uncertain interference is presented. The proposed filtering algorithm is improved by a robust estimation method, wherein both single and constantly changed outliers are considered. In addition, the proposed algorithm was verified by the GPS/INS integrated navigation. The results have shown that the proposed algorithm has better performance than other algorithms.

The conclusions can be summarized as follows:

(1) The adaptive Kalman filtering algorithms were developed in order to reduce the positioning errors, and the proper adaptive factors were selected. The H-infinity filtering algorithm performed well in the GPS/INS integrated navigation system that contained the uncertainties. However, the performance was greatly affected by the outliers.
(2) The integration of the adaptive Kalman filter, the H-infinity filter, and the robust estimation method provided the AHF algorithm, which can address the deviations caused by dynamic model errors and interference. Since the proposed algorithm was verified by real data, a wide application of the proposed AHF algorithm in dynamic navigation and positioning can be expected.

## Figures and Tables

**Table 1:** Root mean square error (RMSE) values of the schemes (m).

Axis	KF	AKF	HF	AHF
X	0.046	0.043	0.039	0.035
Y	0.047	0.045	0.038	0.034
Z	0.058	0.049	0.043	0.042

**Table 2:** RMSE values of the schemes (m).

Axis	KF	AKF	HF	AHF
X	0.129	0.105	0.096	0.078
Y	0.284	0.251	0.204	0.121
Z	0.188	0.160	0.128	0.094

**Table 3:** RMSE values of the schemes (m).

Axis	KF	AKF	HF	AHF
X	0.446	0.282	0.232	0.083
Y	0.494	0.338	0.298	0.128
Z	0.413	0.266	0.222	0.099
